# The circRNA hsa-circ-0013561 regulates head and neck squamous cell carcinoma development via the miR-7-5p/PDK3 axis

**DOI:** 10.1186/s12935-024-03256-x

**Published:** 2024-03-01

**Authors:** Kaisai Tian, Liying Zheng, Tailei Yuan, Xiaoping Chen, Qun Chen, Xiaocheng Xue, Shuixian Huang, Weining He, Mingming Jin, Yi Zhang

**Affiliations:** 1 Postgraduate Training Base at Shanghai Gongli Hospital, Ningxia Medical University, Shanghai, 200135 China; 2 Department of Otorhinolaryngology Head and Neck Surgery, Shanghai Pudong New Area Gongli Hospital, Shanghai, 200135 China; 3 Caolu Community Health Service Center, Pudong New Area, Shanghai, 201209 China; 4 Shanghai Key Laboratory of Molecular Imaging, Shanghai University of Medicine and Health Sciences, Shanghai, 201318 China

**Keywords:** Hsa-circ-0013561, Head and neck squamous cell carcinoma (HNSCC), miR-7-5p, PDK3

## Abstract

**Background:**

Circular RNAs (circRNAs) belong to a class of covalently closed single stranded RNAs that have been implicated in cancer progression. Former investigations showed that hsa-circ-0013561 is abnormally expressed in head and neck squamous cell carcinoma (HNSCC). Nevertheless, the role of hsa-circ-0013561 during the progress of HNSCC still unclear.

**Methods:**

Present study applied FISH and qRT-PCR to examine hsa-circ-0013561 expression in HNSCC cells and tissue samples. Dual-luciferase reporter assay was employed to identify downstream targets of hsa-circ-0013561. Transwell migration, 5-ethynyl-2′-deoxyuridine incorporation, CCK8 and colony formation assays were utilized to test cell migration and proliferation. A mouse tumor xenograft model was utilized to determine the hsa-circ-0013561 roles in HNSCC progression and metastasis in vivo.

**Results:**

We found that hsa-circ-0013561 was upregulated in HNSCC tissue samples. hsa-circ-0013561 downregulation inhibited HNSCC cell proliferation and migration to promote apoptosis and G1 cell cycle arrest. The dual-luciferase reporter assay revealed that miR-7-5p and PDK3 are hsa-circ-0013561 downstream targets. PDK3 overexpression or miR-7-5p suppression reversed the hsa-circ-0013561-induced silencing effects on HNSCC cell proliferation and migration. PDK3 overexpression reversed miR-7-5p-induced effects on HNSCC cell proliferation and migration.

**Conclusion:**

The findings suggest that hsa-circ-0013561 downregulation inhibits HNSCC metastasis and progression through PDK3 expression and miR-7-5p binding modulation.

## Background

Head and neck squamous cell carcinoma (HNSCC) is prevalent and highly aggressive, and its incidence continues to rise. It is predicted that by 2030 there will be about 1.08 million new HNSCC cases diagnosed per year [1]. Malignant SCC is associated with a grim prognosis, increased mortality rates, and a high propensity for lymph node metastasis [2, 3]. Despite significant advancements in treatment, the survival rates of HNSCC patients remain disappointingly low [4]. Further elucidation of the pathogenic molecular mechanisms is critical for novel therapy developments of HNSCC.

circular RNAs (circRNAs) belong to a particular class of ncRNA molecule that forms through non-canonical splicing mechanisms. Reverse splicing connects 3′ end of an exon to 5′ end of the same or upstream exon, forming a closed loop [5]. Former investigations showed that circRNAs like hsa_circ_0016148, hsa_circ_0000264, and hsa_circ_0032822 function importantly in HNSCC progressions [6–8]. In addition, circRNAs can regulate several cancer-related signaling pathways, like those involving microRNA sponges. Although some functions and underlying mechanisms regarding particular circRNAs participated in HNSCC pathogenesis remain known, further investigation is needed to fully elucidate this biology.

In the present study, we found that hsa-circ-0013561 is upregulated in both HNSCC tissues and cells and that it has a negative positive with the migration and invasion of HNSCC cells. Mechanistically, hsa_circ_0013561 can regulation the malignant progression of HNSCC by miR-7-5p/PDK3 signaling pathway. Revealing hsa_circ_0013561 as a potential novel effective target for HNSCC treatment.

## Methods

### Tissue samples

Tissue biopsies of carcinoma and paracancerous samples were taken from three HNSCC patients that were pathologically diagnosed at Pathology Department in Shanghai Gongli Hospital from 2021–2022 and had complete clinical data. Biopsies were paraffin-embedded and formalin-fixed, and the paraffin specimen were used for immunohistochemistry. Ethics Committee in Shanghai Gongli Hospital approved the research. Patients provided consents that informed.

### Fluorescence in situ hybridization (FISH)

We measured hsa_circ_0013561 expression in tissues using FISH. We used Cy3-bound anti-digoxin and FITC-bound anti-biotin antibodies to measure the signal (Geneseed Biotech, Guangzhou, China). We used DAPI to stain nuclei. We used fluorescence Leica microscope to acquire images.

### Cell culture and cell transduction

We purchased Hep-2 cells from Shanghai Phytobiotechnology Company (Shanghai, China). Our team cultured Hep-2 cells in DMEM (Gibco, GrandIsland, NY, USA) medium with 10% FBS (BIOEXPLORER, collected in South America) and 1% streptomycin/penicillin antibiotics under 37 °C and 5% CO_2_.

We constructed lentivirus constructs overexpressing hsa_circ_0013561 (sh-circ0013561) and negative control (NC) vectors. Stable Hep-2 knockdown cells were generated after lentivirus infection. After cell passaging, RNA was extracted to verify infection efficiency.

### Quantitative real-time polymerase chain reaction

We isolated total RNA using RNAfast200 Kit (Fastagen) to measure employing NanoDrop device (Thermo Fisher Scientific, Waltham, Massachusetts, USA). Technician synthesized complementary DNA utilizing cDNA Synthesis Kit (Takara, Ozu, Japan) and synthesized primers through Sangon Biotech (Shanghai, China) using Primer software based on the sequence of each gene in circBase. The primer sequences are: hsa_circ_0013561 5ʹ-GAATGCTGTCCTGTCCTC-3ʹ forward; 5ʹ-TGCCAATCATGGTCAGAG-3ʹ reverse. miR-7-5p Stem-loop primer GTCGTATCCAGTGCAGGGTCCGAGGTATTCGCACTGGATACGACAACAAC. miR-7-5p 5ʹ-CGCGTGGAAGACTAGTGATTTT-3ʹ forward; 5ʹ-AGTGCAGGGTCCGAGGTATT-3ʹ reverse. PDK3 5ʹ-CGCTCTCCATCAAACAATTCCT-3ʹ forward; 5ʹ-CCACTGAAGGGCGGTTAAGTA-3ʹ reverse. β-actin 5ʹ-GGCTGTGCTATCCCTGTACG-3ʹ forward; 5ʹ-CTTGATCTTCATTGTGCTGGGTG-3ʹ reverse. U6 5ʹ-CTCGCTTCGGCAGCACA-3ʹ forward; 5ʹ-AACGCTTCACGAATTTGCGT-3ʹ reverse. β-actin and U6 were utilized as endogenous controls. 2^−ΔΔCt^ method was utilized for calculation of relative gene expression. We made qRT-PCR employing StepOnePlus RT-PCR System (Thermo Fisher Scientific) and SYBR Green PCR Master Mix (Yeasen, Shanghai, China). Thermal cycle conditions for qRT-PCR were: 40 cycles of 0.5 min at 95℃, 10 s at 95 ℃, 30 s at 60 ℃.

### Cell counting kit-8 (CCK8) assay

Following standard protocol, we measured relative cell viability at 1, 2, and 3 days post infection using the CCK-8 (Yeasen, Shanghai, China). We inoculated cells into 96-well plates with 4000 cells per well and five replicates. We next added 10 μl CCK-8 solutions to every well to incubate plates at 37 °C for 1 h in dark. At 0, 24, 48, and 72 h post-seeding, we measured optical density utilizing microplate reader at 450 nm.

### 5-ethynyl-2′-deoxyuridine (EdU) assay

We measured cell proliferation utilizing EdU assay kit (US EVERBRIGHT, Suzhou, China). Our team inoculated Hep-2 cells transduced with normal control vector as well as Hep-2 cell transductions with sh_circ_0013561 vector into 24-well plates (4 × 10^4^ per well) and cultured cells overnight. 10 μl of EdU-labeled medium was added to the 24-well plate and then the plate was incubated at 5% CO_2_ at 37 °C for two hours. We then fixed plates with 4% paraformaldehyde and 0.5% Triton X-100. We added EdU and DAPI staining solutions to wells and imaged cells with fluorescence microscopy (Leica microscope, Shanghai, China). We calculated EdU incorporation rates as the ratio of total EdU-positive cell (red signal) numbers to DAPI-positive cell (blue signal) numbers.

### Colony formation assays

Our team inoculated transduced cells into 6-well plates with density 1000 cells/well in DMEM medium including 10% FBS for one week. Cells were cleaned twice with PBS, fixed with 4% paraformaldehyde for 20 min, and dyed with 0.5% crystal violet for twenty minutes. Colonies comprised of ≥ 50 cells were calculated.

### Apoptosis test

We assessed apoptosis using an apoptosis assay (FITC Annexin V Apoptosis Detection Kit I, BD Pharmingen). We first collected non-adherent cells in the culture supernatant. We cleaned adherent cells twice with PBS and trypsinized them for three minutes. We then mixed cells with culture medium to end the digestion, and centrifuged 1 × 10^6^ cells at 1000 RPM/min for 5 min. We discarded supernatant, and used 100 μl 1 × binding buffer to resuspend cells and transfer them to Eppendorf tubes. 5 μl propidium iodide and 5 μl Annexin V-APC were added to every tube. After mixing, we incubated cells for 15 min at room temperature in dark, followed by adding 500 μl 1 × binding buffer to terminate reactions. Apoptosis was measured utilizing flow cytometry.

### Cell cycle assay

Technician measured cell cycle utilizing a cell cycle assay kit (Cell Cycle and Apoptosis Analysis Kit, Beyotime, China). The same method for cell collection for apoptosis detection was used. A total of 500 μl staining buffer, 10 μl RNase A (50 ×) and 25 μl propidium iodide staining solution (20 ×) was used to resuspend cells after washing with PBS. Cells were transferred to Eppendorf tubes, mixed well, and incubated at room temperature. We assessed the cell cycle using a low loading-speed flow cytometry instrument.

### Wound healing assay

We inoculated control cells and transduced cells into 6-well plates at 3.5 × 10^5^ cells/well to culture them in DMEM medium including 10% FBS. When the confluency reached 90–95%, we created a “scratch” using a 1 ml pipette tip and washed off the detached cells using PBS. Subsequently, serum-free medium was added to culture. At 0, 1, 2 and 3 days post-seeding, we imaged wound area under microscope. Wound area was captured employing Image J software. The cell scratch healing rate was computed as (scratch area at 0 h—scratch area at timepoint)/scratch area at 0 h × 100%.

### Transwell assay

We suspended control and transduced cells in 200 μl serum-free medium with 2 × 10^4^ cells/well. We inoculated cells in Transwell plate upper chamber with 8 μm pore size. In addition, we put 600 μl medium including 15% FBS in lower chamber as a chemotactic agent. One day post-seeding, we fixed Transwell membrane utilizing 4% paraformaldehyde, stained it with 0.1% crystal violet, and viewed 3 random view (100 ×) fields with microscope.

### Western blot analysis

Technician isolated total protein from transduced cells utilizing RIPA Lysis Buffer (RIPA Lysis Buffer Strong, Beyotime, China) through protease inhibitor (Phenylmethanesulfonyl fluoride, Beyotime, China). Technician gained protein concentration through BCA protein kit (Yeasen, Shanghai, China). We separated protein samples (15 μg) using electrophoresis with 10% SDS-PAGE gel to transfer them to PVDF membrane. Next, we blocked membranes with skim milk (5%) for 1 h. Technician incubated primary antibodies on blots at 4 °C overnight on a shaker. Primary antibodies used were: anti-BAX (1:5000, Proteintech, Wuhan, China), anti-BCL-2 (1:5000, Proteintech, Wuhan, China), anti-N-cadherin (1:5000, Proteintech, Wuhan, China), anti-PDK3 (1:1000, Affinity Biosciences, Jiangsu, China), anti-E-cadherin (1:5000, Proteintech, Wuhan, China) and anti-β-actin (1:10,000, Proteintech, Wuhan, China). β-actin was applied as internal reference. On the second day, we incubated blots through peroxidase-labeled secondary antibodies on shaker for one hour. Secondary antibodies used were: goat anti-mouse IgG (1:5000, Biosharp, Anhui, China) and goat anti-rabbit IgG (1:5000, Biodragon, Beijing, China). Then ECL Western Blotting Kit (Vazyme, Nanjing, China) was employed to visualize the protein bands. Lastly, we analyzed bands utilizing Image J software.

### Proteomics

We plated Hep-2 cells at 2.0 × 10^6^ cells in a 10 cm dish. Cells were incubated at 37 ℃ for 1 d to allow for adherence. We lysed cells using RIPA buffer with 1 mM phenylmethanesulfonyl fluoride. Technician measured protein concentration applying a BCA Kit (Yeasen, China). Protein samples were stored at − 80 ℃. A total of 250 μg protein was incubated with 10 mM DTT at 37 ℃ for one hour, followed by alkylation with 50 mM indole-3-acetic acid at 25 ℃ in the dark for 1 h. Then, our team used 1.5 mL prechilled (− 20 ℃) 100% acetone to clean sediment. The sample was centrifuged at 14,000×*g* three times for fifteen minutes. Our group diluted samples with 500 μL NH_4_HCO_3_ and desalted peptides utilizing SPE C18 cartridges, which we lyophilized under a vacuum prior to nano HPLC–MS/MS analyses.

### In vivo experiments

To generate HNSCC mice models, we injected Hep-2 cells (2 × 10^6^) transduced with either NC or sh-circ0013561 viral vectors into nude mice flank. We measured tumor weights and volumes. After 25 days mice were murder and tumor tissues were isolated for further study. Each group have 6 mice. The Animal Ethics Committee in Shanghai Gongli Hospital approved animal experiments.

For tumor metastasis analyses, luminescence-labeled Hep-2 cells were transduced with sh-circ0013561 or NC. We resuspended 2 × 10^5^ cells in PBS and injected them into tail vein for lung metastasis studies, or the pelma for lymphatic metastasis studies. Lung and lymphatic metastases were imaged utilizing in vivo bioluminescence imaging system 4 weeks after injection. We counted metastatic foci in extracted lung tissues based on hematoxylin and eosin (HE) staining. Lymph angiogenesis were detected with CD31 staining.

### Statistical analyses

Statistician assessed differences among groups applying an unpaired t-test, and data are expressed as averages. We considered *p* < *0.05* as statistical significance. Our team performed statistics analysis using GraphPad Prism (GraphPad Inc., San Diego, CA, USA).

## Results

### hsa-circ-0013561 downregulation suppresses HNSCC cell proliferation

**Fig. 1:**
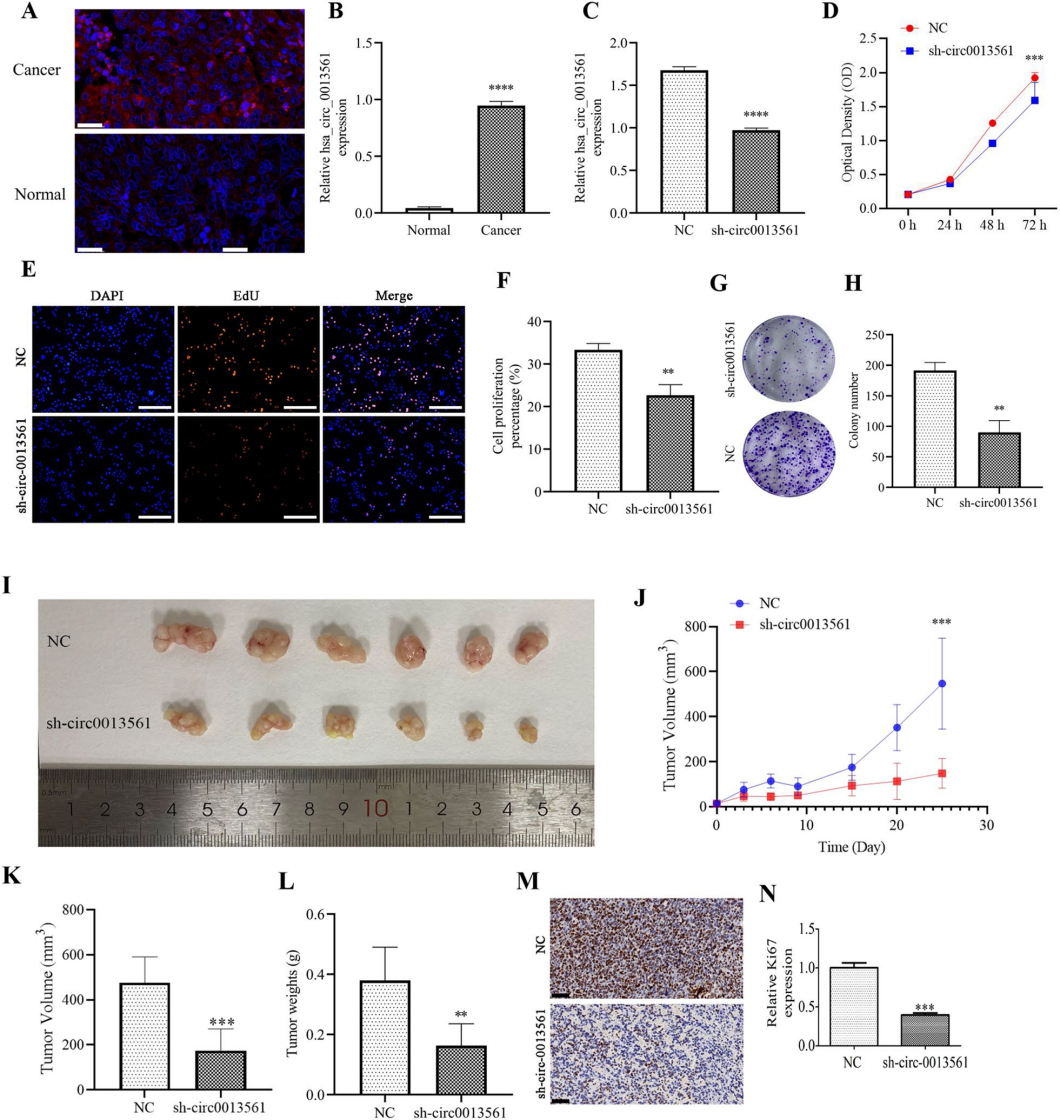
Downregulation of hsa-circ-0013561 inhibits HNSCC cell proliferation and tumor growth. **A**, **B** FISH detection showing the expression and subcellular distribution of hsa-circ-0013561. **C** qRT-PCR detection showing the expression of hsa-circ-0013561 in Hep-2 cells after transduction with NC or sh-circ0013561. ^***^p < 0.001 vs. si-NC. **D** CCK-8 detection showing Hep-2 cell proliferation. ^***^p < 0.001 vs. NC. **E**, **F** EdU assay showing Hep-2 proliferation after transduction with NC or sh-circ0013561. ^**^p < 0.01 vs. NC. **G**, **H** Colony formation assay showing Hep-2 proliferation after transduction with NC or sh-circ0013561. ^**^p < 0.01 vs. NC. All data are expressed as the mean ± SD. **I** Representative images of HNSCC tumor formation in nude mouse xenograft models. **J** Summary of mouse tumor volumes measured every three days. ***p < 0.001 vs. sh-NC. **K**, **L** Tumor volumes and weights measured 25 days post-injection. **p < 0.01, ***p < 0.001 vs. NC. **M**, **N** Immunohistochemistry showing the percentage of Ki67-positive cells. ***p < 0.001 vs. NC. All data are presented as the mean ± SD. All experiments were repeated three times

Hsa-circ-0013561 expression increased in cancer tissues compared to normal tissues, as assessed by FISH, and hsa-circ-0013561 was found to be located mainly in the cytoplasm (Fig. 1A, B). A hsa-circ-0013561 knockdown vector (sh-circ0013561) was transduced into Hep-2 cells, and hsa-circ-0013561 expression was shown to significantly decrease after transduction with sh-circ0013561 (Fig. 1C). CCK-8 (Fig. 1D), EdU (Fig. 1E, F) and colony formation (Fig. 1G, H) data verified that hsa-circ-0013561 knockdown inhibited Hep-2 cell proliferation.

Nude mouse tumor xenograft models utilizing Hep-2 cells showcased that hsa-circ-0013561 downregulation decreased tumor growth by volume and weight (Fig. 1I–L). Immunohistochemistry for Ki67 staining validated that hsa-circ-0013561 downregulation suppressed Ki67 expression in tumor tissues (Fig. 1M, N), suggesting that hsa-circ-0013561 downregulation suppresses HNSCC proliferations.

### hsa-circ-0013561 downregulation promotes HNSCC apoptosis

**Fig. 2:**
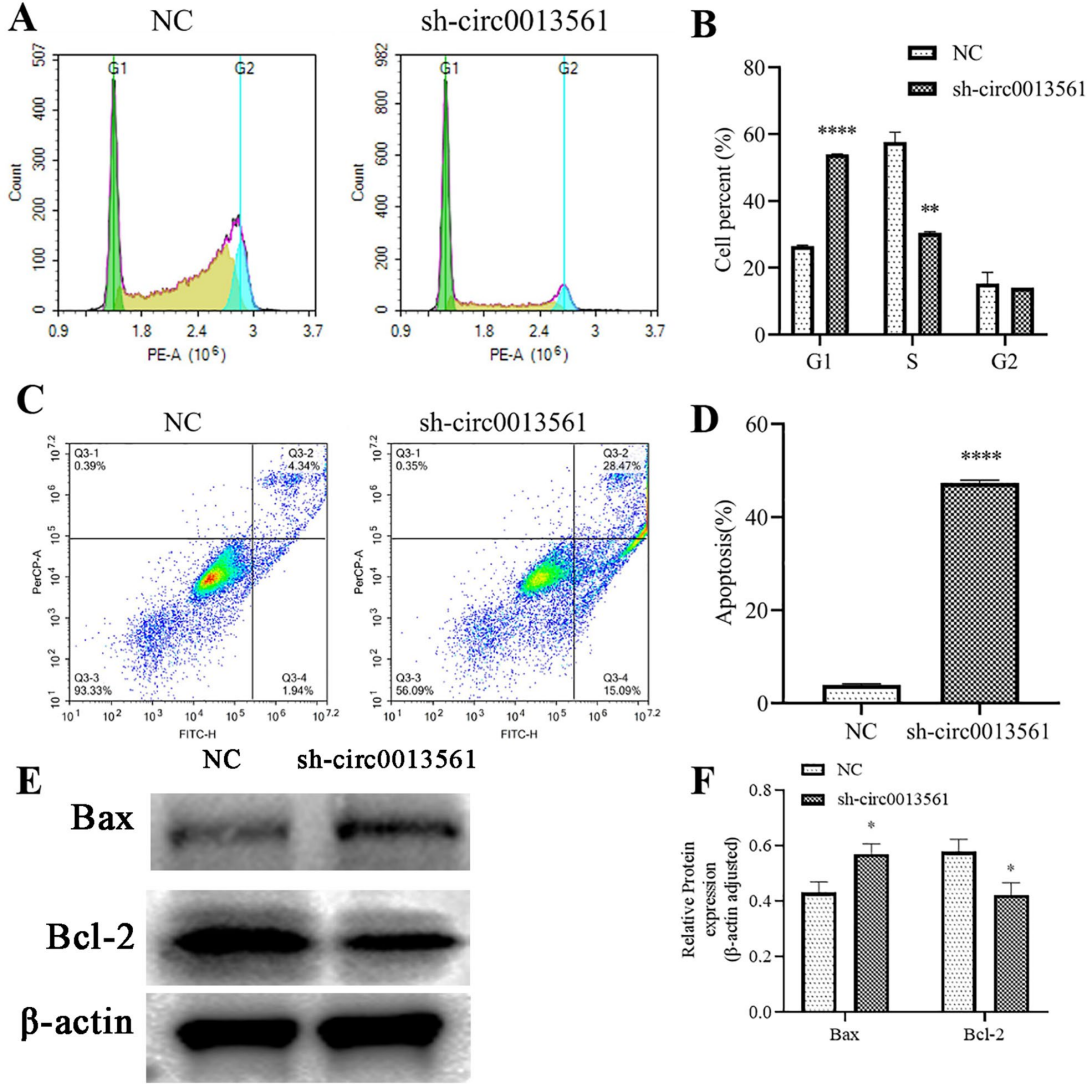
Downregulation of hsa-circ-0013561 promotes HNSCC apoptosis. **A**, **B** Flow cytometry showing the percentage of Hep-2 cells in G1, S, or G2 phase. **p < 0.01, ***p < 0.001 vs. NC. N = 3. **C**, **D** Flow cytometry for apoptosis detection showing the percentage of apoptotic Hep-2 cells. ***p < 0.001 vs. NC. N = 3. **E**, **F** Western blot for apoptotic proteins Bcl-2 and BAX. *p < 0.05 vs. NC. All data are presented as the mean ± SD. All experiments were repeated three times

We stained Hep-2 cells with propidium iodide, and assessed cell cycle utilizing flow cytometry. Compared to NC group, the cell ratios in G0/G1 phase significantly increased in hsa-circ-0013561 knockdown group, suggesting that hsa-circ-0013561 downregulation arrests cells in G1 phase (Fig. 2A, B). Flow cytometry for apoptosis data showcased that hsa_circ_0013561 downregulation promoted Hep-2 cell apoptosis (Fig. 2C, D). Western blotting illustrated that hsa_circ_0013561 downregulation promoted BAX expression, but decreased Bcl-2 expression (Fig. 2E, F). These data support that hsa-circ-0013561 downregulation promotes HNSCC apoptosis.

### Downregulation of hsa-circ-0013561 suppresses HNSCC cell migration

**Fig. 3:**
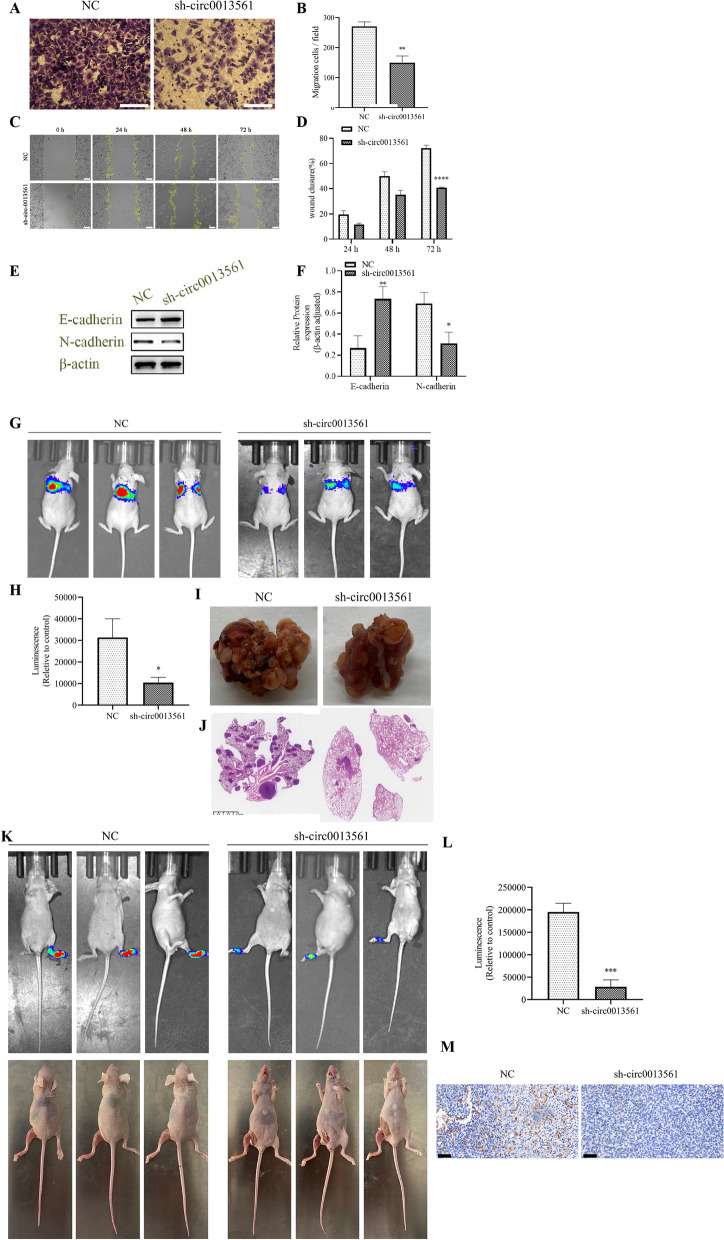
HNSCC cell migration and invasion are inhibited with hsa-circ-0013561 downregulation. **A**, **B** Transwell assay showing the migration of Hep-2 cells after silencing hsa-circ-0013561. ^**^p < 0.01 vs NC. **C**, **D** Wound healing assay showing the invasion of Hep-2 cells after silencing hsa-circ-0013561. ^****^p < 0.0001 vs NC. **E**, **F** Western blot for epithelial-to-mesenchymal transition proteins E-cadherin and N-cadherin. **p < 0.01, *p < 0.05 vs. NC. All data are presented as the mean ± SD. **G**, **H** Live imaging showing HNSCC cell pulmonary metastases in mice. The data are expressed as the mean ± SD. ^*^p < 0.05 vs NC. **I**, **J** The number of metastatic foci in lung tissues were determined using hematoxylin and eosin staining. **K**, **L** Live imaging showing HNSCC cells lymphatic metastases. The data are expressed as the mean ± SD. ***p < 0.001 vs NC. **M** Immunohistochemistry for CD31 staining in lymphatic tissue from mouse models. All experiments were repeated three times

Transwell assay showed that hsa-circ-0013561 downregulation suppressed Hep-2 cell migration (Fig. 3A, B). A wound healing assay illustrated that hsa-circ-0013561 downregulation suppressed Hep-2 cell invasion (Fig. 3C, D). Western blotting validated that hsa_circ_0013561 silencing promoted E-cadherin expression while reducing N-cadherin expression (Fig. 3E, F), suggesting that hsa-circ-0013561 downregulation inhibits epithelial-to-mesenchymal transition of HNSCC.

Live imaging of Hep-2 cell pulmonary metastasis mouse models demonstrated that hsa_circ_0013561 silencing decreased pulmonary metastases and metastatic foci numbers in lung tissues as assessed with HE staining (Fig. 3G–J). Downregulation of hsa-circ-0013561 resulted in decreased CD31 expression (Fig. 3K–M), suggesting that hsa_circ_0013561 downregulation suppressed HNSCC cell invasion.

### miR-7-5p and PDK3 are downstream hsa-circ-0013561 targets

**Fig. 4:**
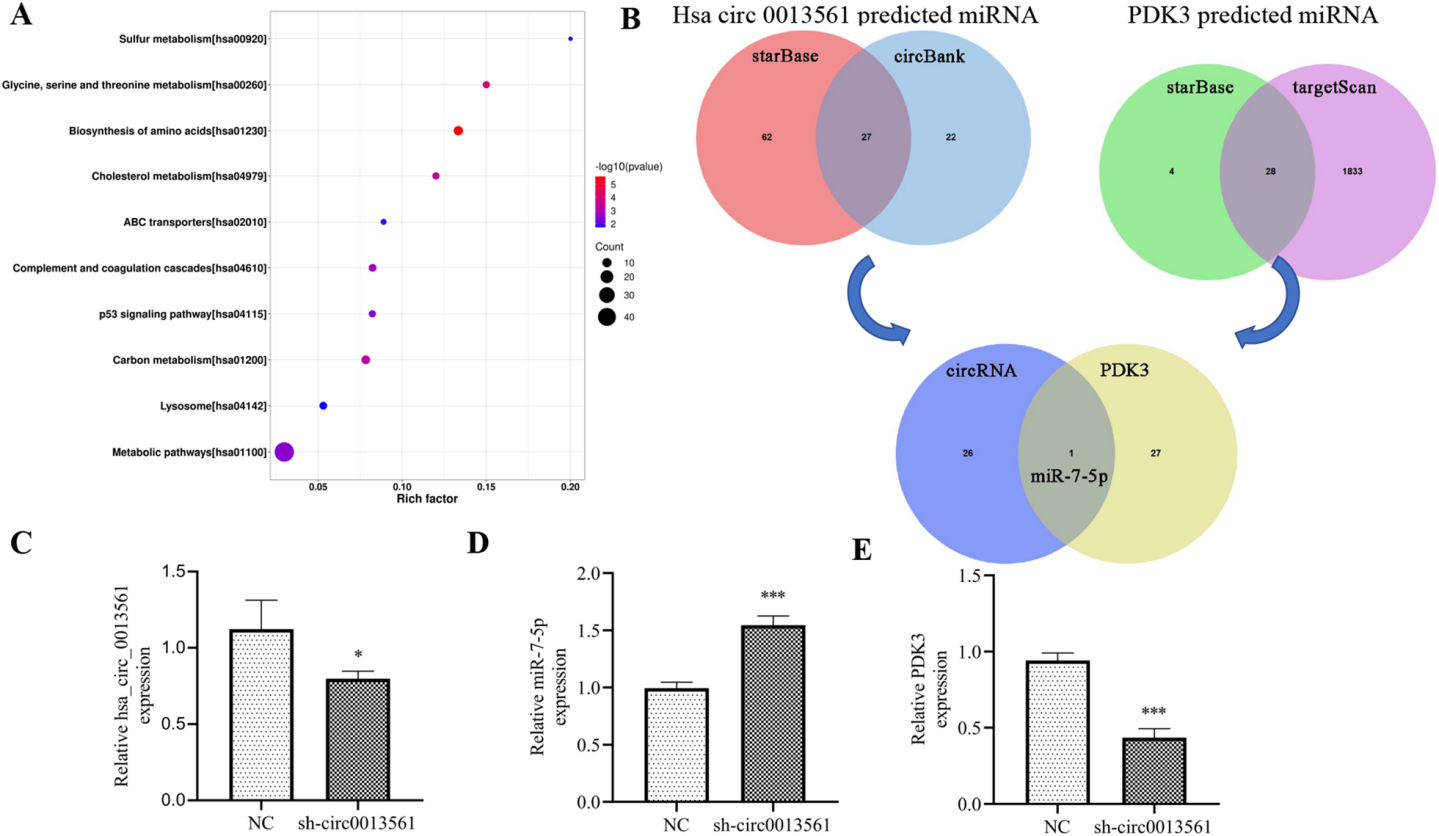
Silencing hsa-circ-0013561 downregulates PDK3 expression but promotes miR-7-5p expression. **A** Proteomics analysis showcased the abnormal protein expression of metabolic pathways after silencet hsa_circ_0013561. **B** Bioinformatics analysis the downstream target of hsa-circ-0013561. **C** RT-qPCR detection show the expression of hsa-circ-0013561, miR-7-5p and PDK3. The data are expressed as the mean ± SD. *p < 0.05, ***p < 0.001 vs NC. All experiments were repeated three times

Proteomics analysis showcased that hsa_circ_0013561 downregulation resulted in abnormal protein expression of metabolic pathways, including PDK3 (Fig. 4A). Bioinformatics analysis suggested that 27 miRNAs could interact with hsa_circ_0013561, and 28 miRNAs could interact with PDK3. Only miR-7-5p was suggested to interact with both hsa_circ_0013561 and PDK3 (Fig. 4B). qRT-PCR data showed that hsa_circ_0013561 downregulation suppressed hsa_circ_0013561 and PDK3 expression, but promoted miR-7-5p expression (Fig. 4C–E).

**Fig. 5:**
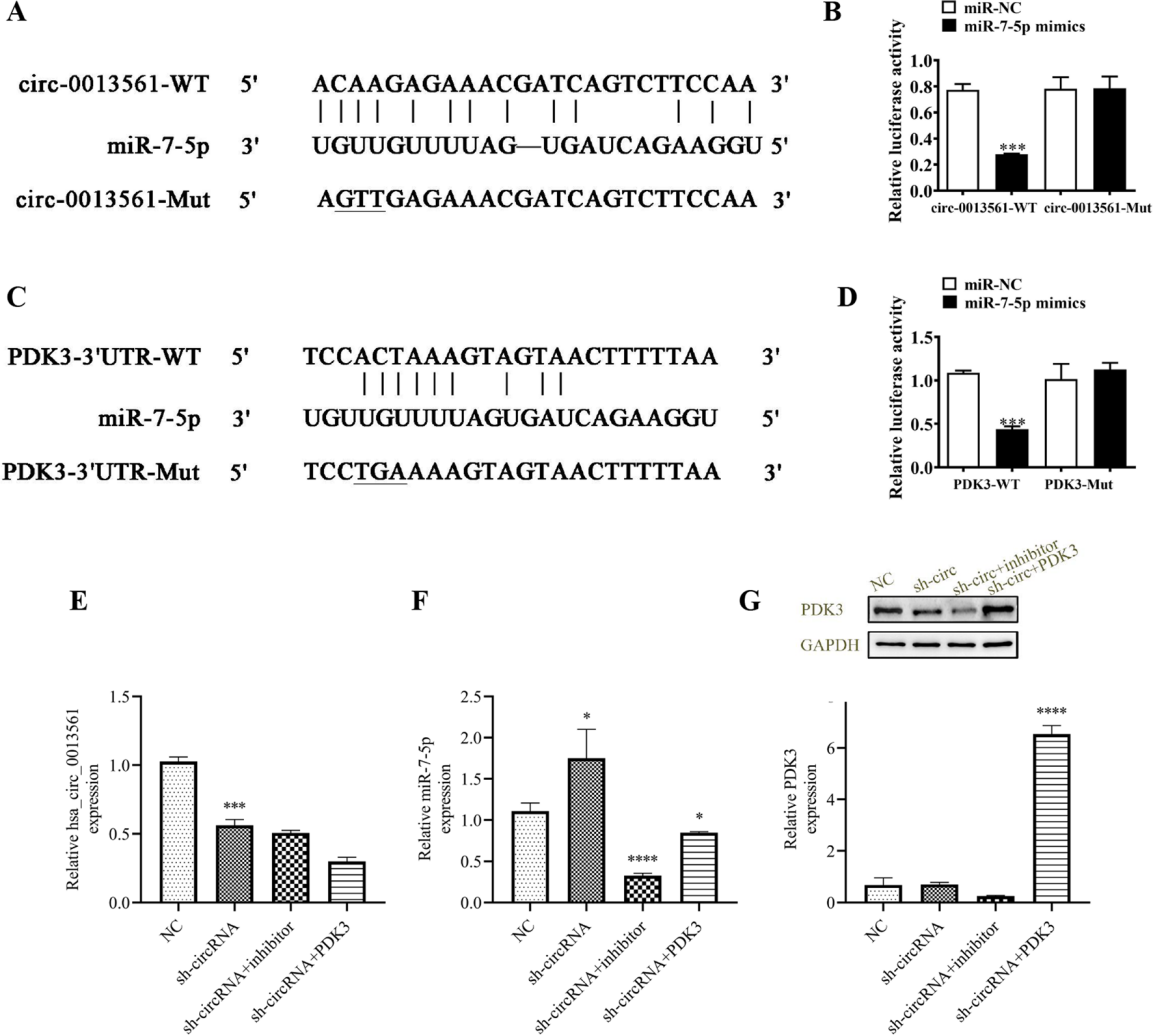
miR-7-5p and PDK3 are downstream targets of hsa-circ-0013561. **A** Prediction of binding sites of miR-7-5p to hsa-circ-0013561. The mutant (MUT) version of hsa-circ-0013561 is presented. **B** Relative luciferase activity 48 h after transduction of Hep-2 cells with miR-7-5p mimic/NC or wild-type (WT)/Mut hsa-circ-0013561-overexpressing lentivirus. **p < 0.01. **C** Prediction of binding sites of miR-7-5p to the 3′-UTR of PDK3. The MUT version of 3′-UTR-PDK3 is shown. **D** Relative luciferase activity determined 48 h after transduction of Hep-2 cells with miR-7-5p mimic/NC or 3′-UTR-PDK3 WT/Mut-overexpressing lentivirus. **p < 0.01. All data are presented as mean ± SD. **E**, **F** qRT-PCR data showing hsa-circ-0013561 and miR-7-5p expression of Hep-2 cells. ^*^p < 0.05, ^***^p < 0.001 vs. NC. **G** qRT-PCR and western blot showing the expression of PDK3. ^****^p < 0.0001 vs. NC. All data are presented as mean ± SD

A luciferase reporter assay verified that miR-7-5p inhibited luciferase activity in WT yet not MUT cells (Fig. 5A, B), suggesting that miR-7-5p is a hsa_circ_0013561 target. Bioinformatics analysis also found that PDK3 is a downstream miR-7-5p target. Luciferase reporter vector was made (Fig. 5C). Luciferase reporter assay outputs demonstrated that miR-7-5p inhibited luciferase activity in WT cells (Fig. 5D), saying that PDK3 is a miR-7-5p target.

Treatment with miR-7-5p inhibitor, or overexpressing PDK3, had no effect on hsa_circ_0013561 expression in Hep-2 cells (Fig. 5E), telling that miR-7-5p and PDK3 are downstream hsa_circ_0013561 targets. PDK3 overexpression did not affect sh-circ-0013561-induced miR-7-5p expression (Fig. 5F). Data support that miR-7-5p is hsa_circ_0013561 downstream target. Silencing of hsa_circ_0013561 reduced PDK3 expression, however, miR-7-5p downregulation reversed sh-circ-0013561 inhibitory effects upon PDK3 expression. Post PDK3 overexpression vector transduction, PDK3 expression significantly incremented (Fig. 5G), suggesting that hsa_circ_0013561 promotes PDK3 expression through binding to miR-7-5p.

### Reversal of HNSCC proliferation and invasion following silencing of hsa-circ-0013561 through PDK3 overexpression or miR-7-5p inhibition

**Fig. 6:**
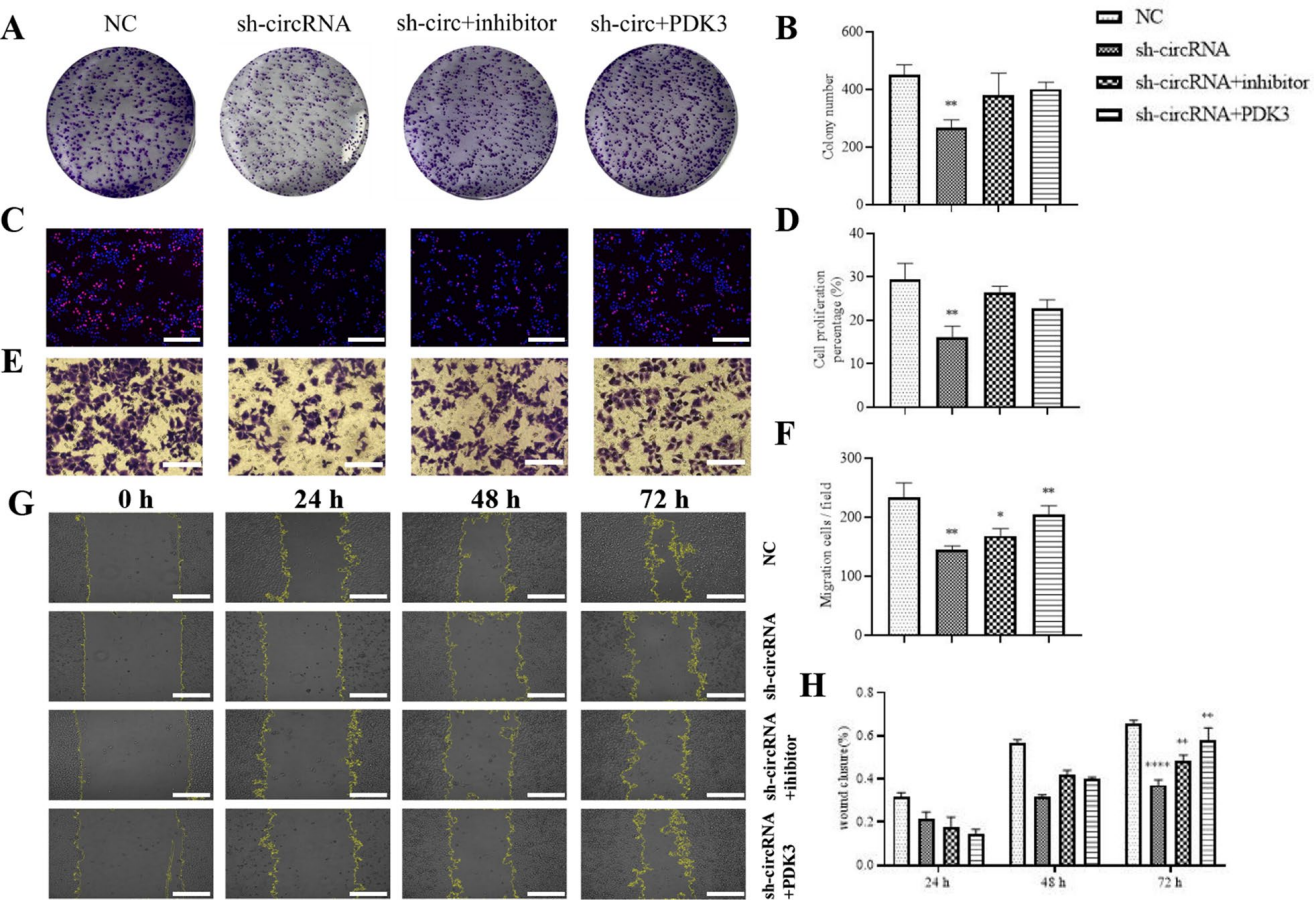
PDK3 overexpression or inhibition of miR-7-5p reverses HNSCC proliferation and invasion after hsa-circ-0013561 silencing. **A**–**D** Colony formation and EdU assays showing Hep-2 proliferation. ^**^p < 0.001 vs NC. **E**–**H** Transwell and wound healing assays showing invasion and migration of Hep-2 cells. ^*^p < 0.05, ^**^p < 0.01, ^****^p < 0.0001 vs NC. All data are expressed as the mean ± SD. All experiments were repeated three times

Colony formation (Fig. 6A, B) and EdU assays (Fig. 6C, D) showcased that PDK3 overexpression or miR-7-5p inhibition restored cell proliferation after silencing hsa_circ_0013561 in Hep-2 cells. Transwell (Fig. 6E, F) and wound healing (Fig. 6G, H) assays validated that PDK3 overexpression or miR-7-5p inhibition restored Hep-2 cell migration and invasion after hsa_circ_0013561 silencing.

### PDK3 overexpression-mediated reversal of HNSCC proliferation and invasion following miR-7-5p overexpression

**Fig. 7:**
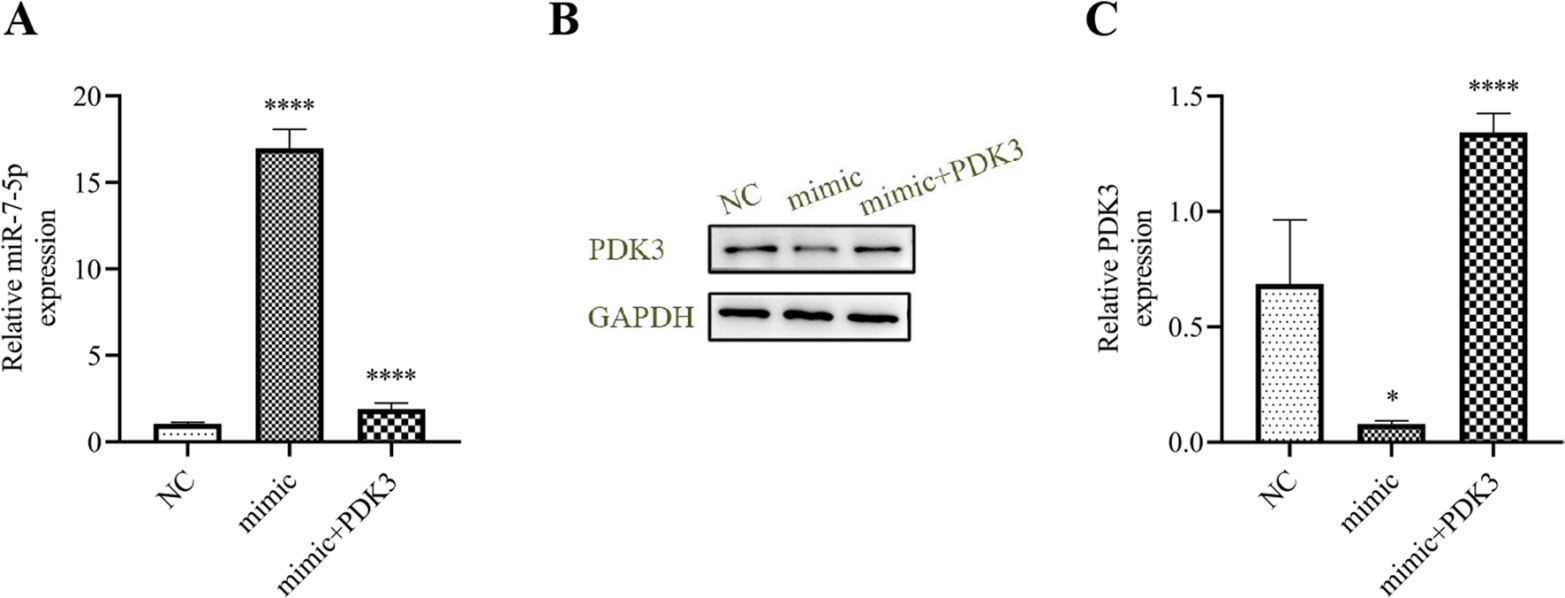
The relationship between miR-7-5p and PDK3. **A** qRT-PCR data showing miR-7-5p expression in Hep-2 cells. ^****^p < 0.0001 vs. NC. **B**, **C** qRT-PCR and western blot showing PDK3expression. ^*^p < 0.05, ^****^p < 0.0001 vs. NC. All data are presented as mean ± SD. All experiments were repeated three times

**Fig. 8:**
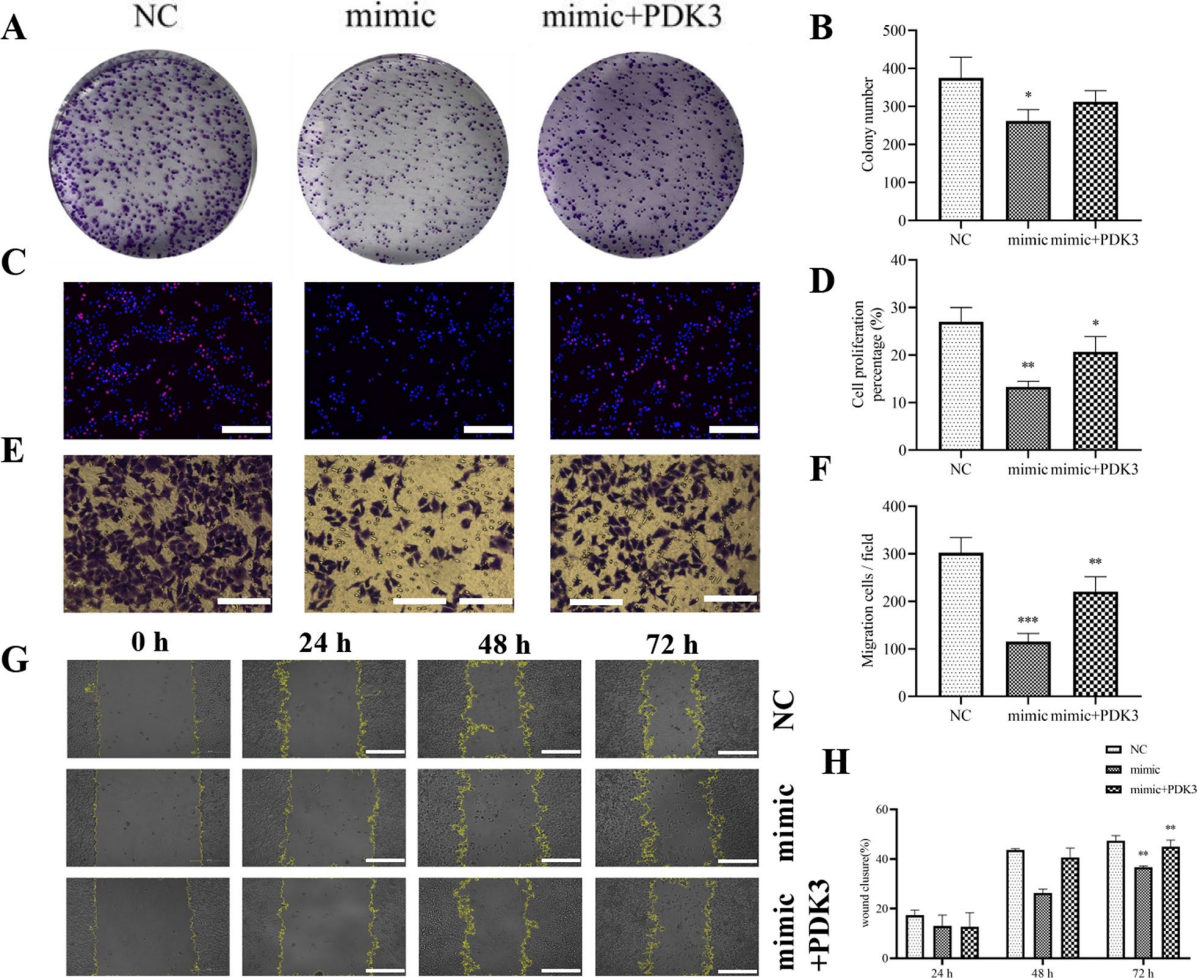
Overexpression of PDK3 reverses HNSCC proliferation and invasion after miR-7-5p upregulation. **A**–**D** Colony formation and EdU assay showing the Hep-2 proliferation. ^*^p < 0.05, ^**^p < 0.001 vs NC. **E**–**H** Transwell and wound healing assay showing Hep-2 cell invasion and migration. ^**^p < 0.01, ^***^p < 0.001 vs NC. All data are expressed as the mean ± SD. All experiments were repeated three times

miR-7-5p overexpression induced miR-7-5p expression, but reduced PDK3 expression, as assessed by qRT-PCR (Fig. 7A). However, overexpression of PDK3 restored and promoted PDK3 expression (Fig. 7B, C). Colony formation (Fig. 8A, B) and EdU (Fig. 8C, D) assays showed that PDK3 overexpression restored Hep-2 cell proliferation after overexpressing miR-7-5p. Transwell (Fig. 8E, F) and wound healing (Fig. 8G, H) assays demonstrated that PDK3 overexpression restored Hep-2 cell invasion and migration following miR-7-5p overexpression. The results advise that PDK3 overexpression reverses HNSCC proliferation and invasion after overexpression of miR-7-5p.

## Discussion

Malignant squamous cell carcinoma, particularly in the case of HNSCC, is characterized by a grim prognosis, significant lymph node metastasis, and elevated mortality rates [9, 10]. The development of numerous human tumors relies heavily on complex signaling networks, which are intricately regulated by various non-coding RNAs such as circRNAs [8, 11]. The specific involvement of circRNAs in the pathogenesis of HNSCC and their underlying molecular mechanisms remain inadequately understood. The present study reveals higher hsa-circ-0013561 expression in HNSCC tissues comparing to normal tissues. We illustrated that hsa-circ-0013561 downregulation promotes cell apoptosis and G1 cell cycle arrest, saying that hsa-circ-0013561 function importantly in HNSCC progression.

Proteomics and bioinformatics data demonstrated that PDK3 and miR-7-5p are downstream hsa-circ-0013561 targets, which was further validated using a luciferase reporter assay. hsa-circ-0013561 downregulation enhanced miR-7-5p expression. Altered miR-7-5p expression was involved in the tumorigenic processes, which is supported by previous reports that miR-7-5p may act as tumor inhibitor in NSCLC, colorectal cancer and pancreatic ductal adenocarcinoma [12–15]. Our data verified that miR-7-5p downregulation reversed sh-circ-0013561 inhibitory effects upon HNSCC proliferation and migration, suggesting that hsa-circ-0013561 silencing inhibits HNSCC progression by enhancing miR-7-5p expression.

More elucidation revealed that PDK3 was miR-7-5p downstream target, which was validated employing luciferase reporter assay. Downregulation of hsa-circ-0013561 inhibited PDK3 expression, while inhibiting miR-7-5p reversed sh-circ-0013561 inhibitory effect upon PDK3 expression. Compared to matched-normal tissues, PDK3 expression is upregulated in HNSCC tissues [16]. PDK3 belongs to PDK family regulating cancer cell metabolic switches [17, 18]. Previous studies have confirmed that PDK3 overexpression enhances cancer cell proliferation [19]. In this study, we found that overexpression of PDK3 restored sh-circ-0013561 inhibitory effects upon HNSCC migration and proliferation. These outcomes unravel that hsa-circ-0013561 silencing suppresses HNSCC progression via promoting miR-7-5p expression and inhibiting PDK3 expression.

In summary, our data supports that hsa-circ-0013561 downregulation suppresses HNSCC proliferation via miR-7-5p/PDK3 signaling axis. The data endorses hsa-circ-0013561 as a candidate diagnostic biomarker, which is a promising therapy target.

## Data Availability

All data generated or analyzed and materials information during the present study are available in the manuscript.
